# A focus on double borane-catalysed sulfenylation of alkynes: two B’s or not two B’s?

**DOI:** 10.1039/d6sc90198k

**Published:** 2026-10-28

**Authors:** Natalia A. F. Atherley, Andrew R. Jupp

**Affiliations:** a School of Chemistry, University of Birmingham Edgbaston Birmingham B15 2TT UK a.jupp@bham.ac.uk

## Abstract

In this article, we highlight the recent report of Melen, Baik and co-workers (Mu-Hyun Baik, Rebecca L. Melen *et al.*, *Chem. Sci.*, 2026, **17**, 11682, https://doi.org/10.1039/d6sc01123c) on the B(C_6_F_5_)_3_-catalysed annulative sulfenylation of alkynes to form a range of 5- and 6-membered heterocycles.

## Abstract 2

This article highlights recent work by Melen, Baik and co-workers on the borane-catalysed synthesis of 5 and 6-membered sulfenylated heterocycles *via* a thiirenium ion intermediate, with a focus on the role of two Lewis acid catalysts in the key step.

## Graphical abstract

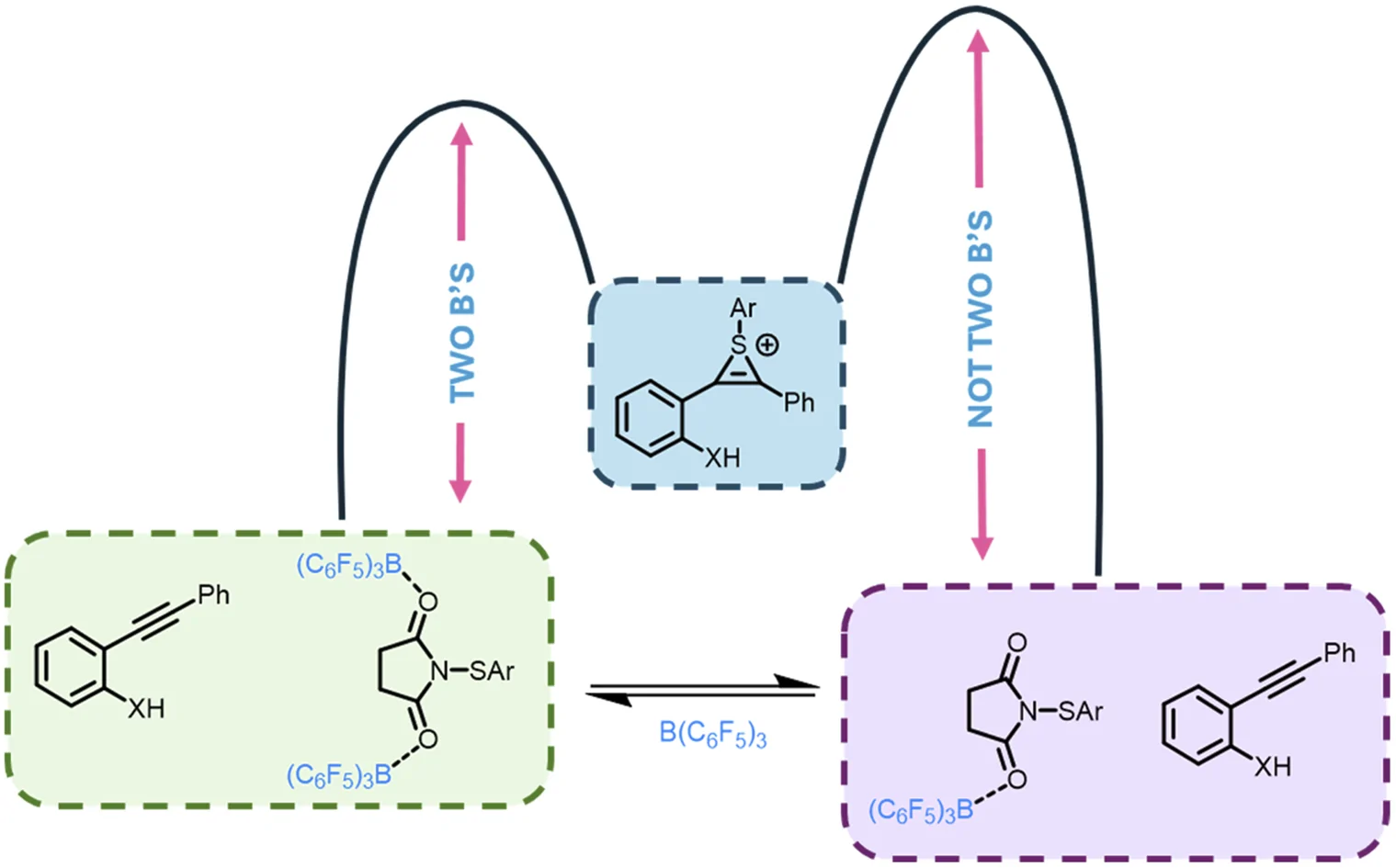

There is a growing interest in the development of earth-abundant main-group catalysts as alternatives to transition metals for the synthesis and functionalisation of industrially relevant compounds. In pursuit of this effort, borane-based reagents appear at the forefront of the literature owing to their strong Lewis acidity. Unlike transition metals which typically activate bonds in a synergic fashion through metal–ligand bonding and back-bonding, boron relies on its empty p_*z*_ orbital to accept electron density. In particular, tris(pentafluorophenyl)borane (B(C_6_F_5_)_3_) is ubiquitous owing to its high electrophilicity and high thermal stability. It initially garnered interest as an initiator in olefin polymerisation in the Ziegler–Natta process,^1^ and is probably best known today as a component of Stephan’s frustrated Lewis pairs (FLPs) for the activation of small molecules,^2,3^ which was conceptually preceded by Piers’ hydrosilylation of carbonyls.^4^ B(C_6_F_5_)_3_ has also found applications in organic chemistry as a Lewis acid in its own right, such as in cyclisation to form heterocycles, C–H activation, and dehydrogenation.^5–8^

In the highlighted article (https://doi.org/10.1039/d6sc01123c),^9^ Melen, Baik and co-workers report the most recent results from their on-going efforts^8,10^ to use B(C_6_F_5_)_3_ as a catalyst with *N*-(arylthio)succinimides (1) to promote the sulfenylation of unsaturated molecules to produce useful heterocycles (Fig. 1). In this protocol, 2-alkynyl-phenols (2, X = O) and protected 2-alkynyl-anilines (2, X = NTs) could be efficiently converted to benzo-*b*-furans and 3-sulfenyl indoles, respectively (4). The methodology could be extended to convert propargyl ethers (3, Y = O), 1,4-diphenylbut-1-yne (3, Y = CH_2_), and a protected propargylaniline (3, Y = NTs) to produce analogous 6-membered rings (5) in good yields. The reactions were tolerant to a range of functional groups. Diyne derivatives of aniline and phenol could undergo annulative π-extension (APEX) reactions to be converted to sulfenylated polyaromatic heterocycles, which exhibited interesting fluorescence properties. Overall, the authors have presented a new metal-free protocol for accessing a diverse range of biologically relevant heterocycles.

**Fig. 1:**
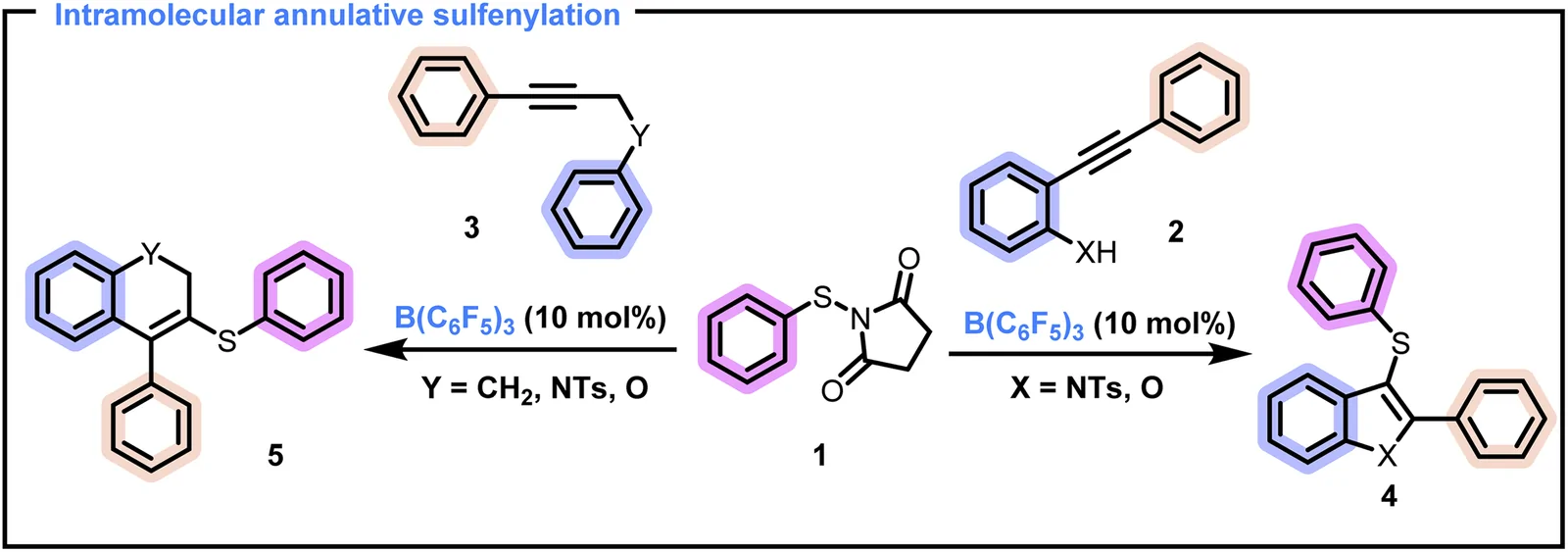
Intramolecular annulative reactions of internal alkynes 2 or 3 to form 5- and 6-membered sulfur-bearing heterocycles, respectively, catalysed by B(C_6_F_5_)_3_.

The proposed mechanism involves the Lewis acid activation of the thiosuccinimide. This activates the SAr fragment to attack from the alkyne moiety, which generates the key thiirenium intermediate (Fig. 2), by analogy with the previous reports on thiiranium intermediates from alkenes.^8,10^ However, in contrast with the previous reports, this highlighted article describes the interesting mechanistic insight that the reaction proceeds by double Lewis acid activation, *i.e.* two B(C_6_F_5_)_3_ molecules activate the thiosuccinimide (one coordinates to each carbonyl oxygen atom, Fig. 2). This was determined using Density Functional Theory (DFT) calculations on a model phenol-derived product, which revealed that the activation barrier to the thiirenium intermediate was higher from singly coordinated thiosuccinimide (23.96 kcal mol^−1^) than from the doubly activated species (21.77 kcal mol^−1^).

**Fig. 2:**
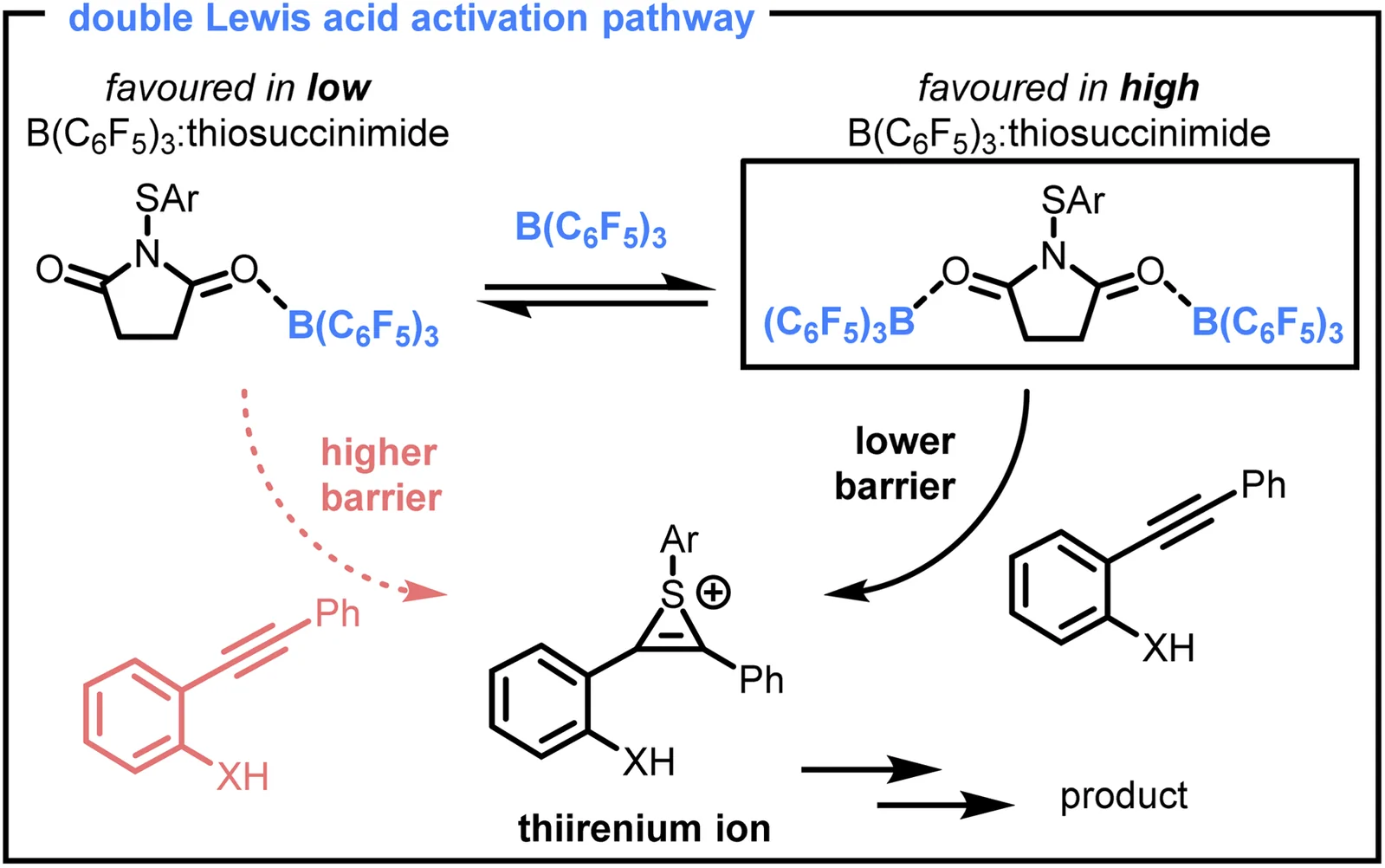
Key intermediates of the proposed mechanism, showing double Lewis acid activation of the thiosuccinimide to generate the thiirenium ion.

Experimental observations corroborate this double Lewis acid activation pathway. Titration of thiosuccinimide against B(C_6_F_5_)_3_ found the concentration of the doubly coordinated species was highest in a high [B(C_6_F_5_)_3_] : [thiosuccinimide] regime, as would be predicted. The proposed mechanism simplistically indicates the reaction is second order in [B(C_6_F_5_)_3_], and gratifyingly when the concentration of the borane catalyst is doubled from 10 mol% to 20 mol%, the concentration of the doubly activated intermediate increases by over four-fold, and the rate of reaction increases significantly. The overall reaction is further complicated by the fact that the succinimide byproduct can bind relatively strongly to the borane catalyst, which means as the reaction progresses the effective concentration of B(C_6_F_5_)_3_ decreases. This disfavours the formation of the doubly activated species, and so it is plausible that at some point during the reaction the singly activated pathway becomes dominant. But the authors have convincingly shown that the reaction does proceed, at least initially, with two B(C_6_F_5_)_3_ molecules activating the substrate in tandem.

This finding provokes the question of how common a phenomenon this double Lewis acid pathway is, or how common could it be if high enough catalyst loadings are used to overcome equilibrium effects and access the doubly activated intermediates. There are plenty of examples of two equivalents of simple Lewis acid catalysts being added to promote organic transformations,^11^ often due to decomposition or byproducts irreversibly binding to one equivalent of the Lewis acid, but it is much rarer to demonstrate that the reaction is promoted by two Lewis acids binding to and activating a single substrate.^12,13^ If the double activation is a possibility for a given substrate, then it could be worth considering using a high loading of catalyst to probe this. In the highlighted work,^9^ there is a fine balance of the Lewis acid needing to be strong enough to (doubly) activate the thiosuccinimide, but not so strong that it is completely sequestered by succinimide byproduct. BF_3_·OEt_2_ was a less effective Lewis acid than B(C_6_F_5_)_3_ for the synthesis of the tested sulfenylindole product (36% yield *vs.* 88%, respectively, at 10 mol% catalyst loading); but the simplicity and ready availability of the former might make it an attractive catalyst choice if higher loadings give rise to significant rate enhancements by unlocking the double activation pathway.

This focus article has centred on an elegant and thorough study that can generate a range of 5- and 6-membered heterocycles bearing a sulfur substituent. Beyond the use of the products themselves, it has also demonstrated the crucial role of two Lewis acids acting in concert on a substrate to drive a reaction. These results will help design optimum Lewis acid catalysts for future organic transformations.

## Author contributions

NAFA and ARJ co-wrote the manuscript.

## Conflicts of interest

There are no conflicts to declare.

## Data Availability

There are no additional data associated with this article.
